# Enhancing plant disease detection through deep learning: a Depthwise CNN with squeeze and excitation integration and residual skip connections

**DOI:** 10.3389/fpls.2024.1505857

**Published:** 2025-01-23

**Authors:** Asadulla Y. Ashurov, Mehdhar S. A. M. Al-Gaashani, Nagwan A. Samee, Reem Alkanhel, Ghada Atteia, Hanaa A. Abdallah, Mohammed Saleh Ali Muthanna

**Affiliations:** ^1^ School of Automation, Chongqing University of Posts and Telecommunications, Chongqing, China; ^2^ School of Resources and Environment, University of Electronic Science and Technology of China, Chengdu, Sichuan, China; ^3^ Department of Information Technology, College of Computer and Information Sciences, Princess Nourah bint Abdulrahman University, Riyadh, Saudi Arabia; ^4^ Department of International Business Management, Tashkent State University of Economics, Tashkent, Uzbekistan

**Keywords:** deep learning, plant disease detection, convolutional neural network, squeeze and excitation (SE) blocks, residual skip connection

## Abstract

This study proposes an advanced method for plant disease detection utilizing a modified depthwise convolutional neural network (CNN) integrated with squeeze-and-excitation (SE) blocks and improved residual skip connections. In light of increasing global challenges related to food security and sustainable agriculture, this research focuses on developing a highly efficient and accurate automated system for identifying plant diseases, thereby contributing to enhanced crop protection and yield optimization. The proposed model is trained on a comprehensive dataset encompassing various plant species and disease categories, ensuring robust performance and adaptability. By evaluating the model with online random images, demonstrate its significant adaptability and effectiveness in overcoming key challenges, such as achieving high accuracy and meeting the practical demands of agricultural applications. The architectural modifications are specifically designed to enhance feature extraction and classification performance, all while maintaining computational efficiency. The evaluation results further highlight the model’s effectiveness, achieving an accuracy of 98% and an F1 score of 98.2%. These findings emphasize the model’s potential as a practical tool for disease identification in agricultural applications, supporting timely and informed decision-making for crop protection.

## 1 Introduction

The global agricultural landscape is presently confronted with an urgent problem: the need for increased food security and sustainable agricultural practices. The imperative to combat the prevalence of plant diseases, which can significantly reduce crop yields and threaten food production, is central to this effort. Plant diseases are a major concern for the agricultural industry and require effective solutions for early detection and intervention Bouguettaya et al. (2023); Prakash et al. (2023); Chen et al. (2020). Despite its critical contribution to economic stability and global food security, agriculture is perpetually threatened by plant disorders that result in significant crop yield reductions Ferehan et al. (2022). The detrimental effects of these diseases on agricultural output, food security, and livelihoods underscore the urgent requirement for efficient plant disease control.

Detection of plant diseases has historically been performed through laboratory analyses and manual inspections. Although precise, these approaches require significant effort, consume considerable time, and are not feasible for large agricultural enterprises. The inadequacies of traditional methods emphasize the need for more sophisticated, automated approaches to enhance the identification and control of diseases. In recent times, the field of plant disease detection has been profoundly transformed by the implementation of deep learning, which has substantially improved both precision and efficiency. In order to identify various plant diseases from images, convolutional neural networks (CNNs) and other contemporary methods have demonstrated great promise by automating the detection process and decreasing reliance on human expertise. These developments have facilitated the expeditious and reliable management of diseases. Expanding upon the advancements mentioned above, this study presents a novel methodology that employs a depthwise CNN that has been improved with a residual skip connection block and a squeeze and excitation block (SE). The model is trained using an extensive dataset comprising plant images, which includes a diverse array of diseased and healthy leaf categories. The ultimate goal of this integration is to provide a reliable and accurate approach to the automated detection of plant diseases, thereby improving both crop protection and yield. Our research seeks to tackle pragmatic obstacles in tangible agricultural environments. It makes a valuable contribution to the overall goal of guaranteeing global food security through the protection of crop well-being and yield. The high accuracy rates and F1 scores attained for disease classification demonstrate the applicability of this model in agricultural settings. Globally, ensuring food security is a top priority Mehrabi et al. (2022); Chelloug et al. (2023), and the capacity to detect and manage plant diseases autonomously contributes substantially to this effort. Furthermore, this study serves as a foundation for future research and development in the fields of plant disease diagnosis and agricultural technology.

## 2 Literature review

The agricultural industry worldwide is under increasing pressure to strengthen food security, all while promoting environmentally sustainable farming methods. The pressing need to mitigate the adverse consequences of plant diseases, which have the potential to significantly reduce agricultural yields and compromise food supply chains, is a critical component of this challenge. The prerequisite for effective strategies for early identification and timely intervention is underscored by the substantial risk to agricultural productivity posed by the prevalence of plant diseases Martinelli et al. (2015); Jones and Naidu (2019). This section provides an in-depth examination of pertinent studies, organized into several key subsections, such as DL applications in plant disease detection, architectural advancements in CNN, challenges and special issues in plant disease detection, and real-world agricultural implications and future directions. The amalgamation of transfer learning and fine-tuning signifies a substantial progression in the use of DL for the identification of plant diseases. Ju et al. (2022) presents a novel approach to categorizing jujubes that takes advantage of transfer learning and CNN to tackle the difficulties associated with sorting agricultural products by quality. Through the incorporation of the SE module and the application of triplet loss and center loss functions, the model is capable of accurately identifying defects in jujubes. By conducting training on a limited dataset supplemented with real production line images, the model exhibits a remarkable accuracy rate of 94.15% and resilience in intricate settings, as verified through the utilization of heatmap visualization and comparison with alternative models. Al-Gaashani et al. (2023) introduced a self-attention network (SANet) based on the ResNet50 architecture, achieving a test set accuracy of 98.71% for rice disease classification. This demonstrates the potential of attention mechanisms in enhancing feature representation and improving efficiency in agricultural disease management. Similarly, their exploration of attention-embedded residual networks for tomato leaf disease detection provided evidence of enhanced feature extraction capabilities and notable improvements in model performance Al-gaashani et al. (2022), underscoring the relevance of attention mechanisms in precision agriculture. Nevertheless, whereas these studies demonstrate considerable progress, they fail to address crucial problems. The computational trade-offs of attention-based designs in resource-limited agricultural environments are yet inadequately examined. The incorporation of these technologies with hybrid architectures or real-time monitoring systems is rarely discussed. The suggested model aims to address these deficiencies by utilizing an innovative amalgamation of lightweight design and effective attention mechanisms, offering a scalable solution with enhanced accuracy and adaptability for agricultural disease management. Pavithra Kalpana and Vigneswaran, (2023) proposed an approach to automate plant disease detection and classification using DL specifically designed for precision agriculture. The DL-APDDC algorithm is specifically designed to detect and classify plant diseases that affect the foliar and reproductive regions. These regions are extracted using U2Net-based background removal, followed by feature extraction utilizing the SqueezeNet model with hyperparameters fine-tuned by the Adam optimizer. The XGBoost classifier concludes with the classification of diseases. Experimental validation on benchmark datasets demonstrates that DL-APDDC outperforms alternative methods for precision agriculture-based automated disease detection. The introduction of separable depth convolutions has transformed CNN architectures. These networks offer both computational efficiency and precision. This Minhaz Hossain et al. (2022) reference investigated the substantial implications that plant diseases have on agricultural production and evaluated the potential of using DL techniques to detect plant diseases effectively. For the purpose of tackling memory limitations, particularly on mobile devices, a model based on depthwise separable convolution is trained. The training is done using a dataset that consists of 2880 images of tomato plants. In identifying nine tomato leaf diseases, the reduced MobileNet model demonstrates good performance, achieving an accuracy of 98. 31% and an F1 score of 92. 03%. Moreover, it strikes a balance between computational efficiency and parameter scale.

In Tang et al. (2020) this research, authors presented a mobile device-optimized lightweight CNN model for the diagnosis of grape maladies. The model is constructed using the channel-wise attention mechanism and is constructed using ShuffleNet V1 and V2 backbones with SE blocks to enhance its performance. The accuracy of the proposed technique is 99.14% when evaluated on a dataset consisting of 4,062 images of grape leaves, including both diseased and healthy subjects. Furthermore, the size of the model is substantially reduced from 227.5 to 4.2 MB, thereby showcasing its efficacy in terms of practical implementation on mobile devices. For diagnosing grape diseases on mobile devices, the proposed lightweight CNN with a channel-wise attention mechanism provides reduced model size and improved accuracy. However, potential drawbacks include computational inefficiency and the requirement for specific dataset characteristics to be met in order to achieve optimal performance, as well as trade-offs between model complexity and efficiency. Chen et al. (2021) proposed the application of deep CNN to transfer learning, specifically integrating MobileNet with an SE block, to improve the identification of plant diseases. The SE-MobileNet model, which was developed using two phases of transfer learning, demonstrated remarkable accuracy rates of 99.78% when applied to clear background datasets and 99. 33% when applied to heterogeneous background datasets. These results highlight the model’s efficiency and efficacy in comparison to currently available approaches. However, additional research is necessary to address potential constraints that may manifest in situations involving dynamic or intricate contexts. Shah et al. (2022) used CNNs with Residual Teacher/Student architecture, which is an improvement over the Teacher/Student model and facilitates the diagnosis of plant diseases. ResTS improves disease categorization by leveraging the representation conveyed between the two classifiers through reciprocal training. This enables visualization of dominant areas in images. In this investigation, authors reached in terms of accuracy 99.1% and F1 score 97.2%. In R et al. (2020), this study investigated the use of CNNs for automated plant disease detection and proposed two architectures: one that incorporates an attention mechanism and the other that uses residual learning. When evaluated on the Plant Village Dataset using 5-fold cross-validation, the models attain a remarkable accuracy of 98%. The benefit resides in the automated feature extraction performed by CNNs, which improves the accuracy of classification. However, significant computational resources may be required, and there is a risk of overfitting when deep models are trained on limited datasets. The symptoms and manifestations of plant disorders vary considerably between species. Implementing disease detection models that can be applied to various plant species and diseases presents a considerable challenge. Studies such as those of Golhani et al. (2018) in order to detect plant diseases, this article provided an exhaustive examination of advanced neural network (NN) methods related to the analysis of hyperspectral data. It comprises an exhaustive analysis of NN mechanisms, models, and classifiers utilized in the processing of imaging and non-imaging hyperspectral data. Particular emphasis is placed on the hybridization of neural networks (NN) with hyperspectral data, specifically in the context of early disease detection. In this regard, the Spectral Disease Index (SDI) holds considerable importance. In addition, the paper examines current challenges and prospective trends in hyperspectral data analysis and discusses NN techniques designed to accelerate the development of SDI. In Garcia and Barbedo (2019), authors examined an innovative methodology for the automated detection of plant diseases by analyzing specific lesions and areas as opposed to the entire foliage. By directing attention toward particular areas, the variability of the data is enhanced without the need for supplementary images, thus facilitating the detection of numerous disorders on a single leaf. However, complete automation still needs to be improved by requiring manual segmentation of symptoms. Notwithstanding this limitation, the methodology shows encouraging results, as evidenced by average accuracies that are 12% higher than those obtained with the original images. It should be noted that despite the inclusion of ten diseases, all crops maintained accuracy above 75%. Although the database may not encompass all practical scenarios, the results of this study highlight the efficacy of deep learning methods in the identification and classification of plant diseases, especially when ample data is accessible. Han et al. (2024) investigates the impact of geographic origin on the chemical composition of garlic using mid-infrared and ultraviolet spectroscopy, utilizing advanced preprocessing methods including Multiple Scattering Correction (MSC), Savitzky–Golay Smoothing (SG Smoothing), and Standard Normalized Variate (SNV). Machine learning models, such as XGBoost, SVC, RF, and ANN, were utilized on the spectral data, attaining up to 100% accuracy in determining the origin of garlic following data fusion. The findings highlight the efficacy of integrating spectral data with machine learning to precisely ascertain the origin of agricultural products, providing significant insights for analogous uses in plant disease identification and categorization through deep learning models. Comparable techniques, including Depthwise CNNs integrated with SE modules and residual skip connections, can improve plant disease detection by adeptly capturing intricate spatial features in plant imagery, resulting in enhanced classification and diagnosis accuracy. Spanaki et al. (2022) reviewed a comprehensive analysis of the latest developments in agricultural technology, classifying a variety of AI-powered methods and their implementation in intelligent, environmentally conscious, and productive agriculture. By conducting a systematic review of the literature, this study provides an all-encompassing resource that helps to understand and delineate the dynamic agricultural technology domain. Establishes a research agenda for forthcoming advancements in agricultural operations while informing stakeholders, including farmers and academics, that AI-driven agricultural technology research is still in its infancy within operations research. Zhang et al. (2024) proposed a flexible visible (Vis)/near-infrared (NIR) real-time sensing system (FVN) for monitoring chilling injury (CI) in bananas during transportation. Based on a multiple linear regression (MLR) model, the system achieves high accuracy in predicting color space parameters, with an R**^2^** p of 0.97 for a* and RPD values exceeding 2.5 for L* and b*. A self-developed classification prediction model (SCP) demonstrated 98.3% and 95.5% accuracy in predicting the occurrence and duration of CI. The FVN system outperforms traditional methods in power consumption, cost, and real-time applicability, significantly reducing banana waste and promoting sustainable production in the supply chain. While it offers advantages in accuracy, cost-effectiveness, and sustainability, limitations include potential sensitivity to environmental variations, the need for further validation across different fruit types, and challenges in real-time integration in complex environments. Jararweh et al. (2023) investigated some statistics and researched them in the real world and the future directions of prediction of plant diseases with AI. According to their investigation, significant progress has been made in the agricultural sector, which is vital to the expansion of the global economy, increasing the capacity and effectiveness of agricultural operations. Given the projected global population growth of 9.6 billion by 2050 and 8.5 billion by 2030, an unprecedented demand for agricultural products and food has emerged, requiring a substantial increase of 70 percent in food production. However, innovative smart agriculture solutions are imperative due to obstacles such as restricted agricultural land, water scarcity, climate change, and changing environmental circumstances. These challenges are addressed by intelligent and precise agriculture, which uses technologies such as IoT, sensors, robotics, AI, intelligent supply chains, big data analytics, and blockchain. The IoT functions as the fundamental infrastructure for the integration of these technologies, facilitating proactive decision-making and diminishing the need for manual labor; consequently, it improves productivity and efficiency.

This comprehensive review of the literature emphasizes the advancing domain of plant disease detection, illustrating the capabilities of deep learning, architectural advancements, and multimodal strategies to improve disease identification. Notwithstanding these gains, the area persists in confronting significant obstacles, including generalization, data imbalance, and limits related to real-world implementation. Addressing these concerns is essential for converting research into practical solutions that protect crop health and enhance global food security.

## 3 Materials and methods

This section presents a comprehensive description of the materials, dataset, and methodologies used in our investigation to construct a precise model to detect plant diseases. We used a depthwise CNN that is integrated with a residual skip connection block and the SE block.

### 3.1 Dataset

**Figure 1:**
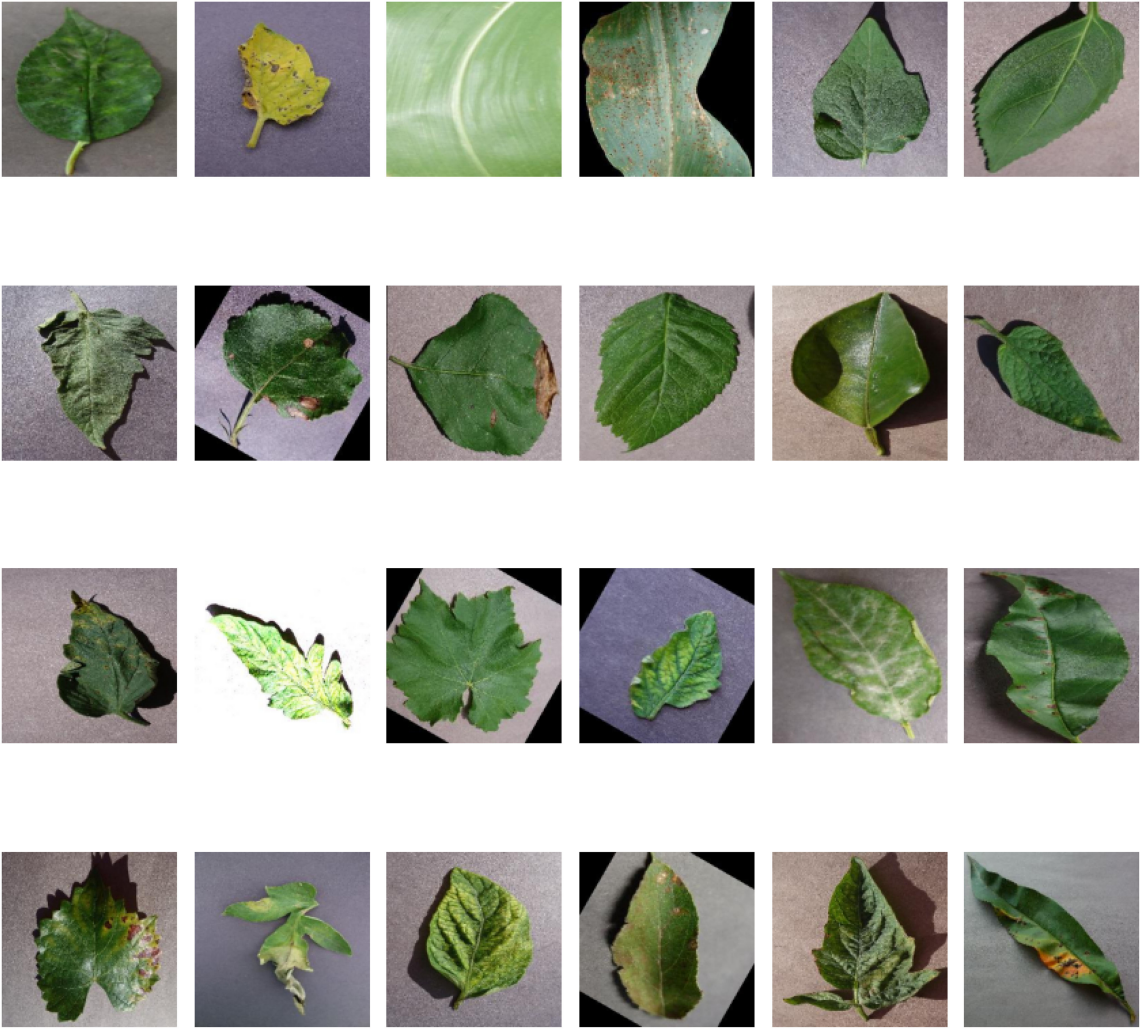
Original samples of the crop disease and healthy leaves images of the dataset.

The findings of our research are derived from an extensive collection of plant images that includes an assortment of plant species and disease classifications. A broad spectrum of plant diseases was captured in the dataset, which was meticulously curated to ensure inclusivity and diversity. Each RGB image in the dataset is annotated with the class and species of the corresponding plant disease. The dataset used in this study is publicly available and has been sourced from Mohanty et al. (2016). The representative samples and comprehensive characteristics of the dataset selected for this study are depicted in **Figure 1** and **Table 1**.

**Table 1:** Details of the dataset.

№	Class Name	Number of Images
0	Tomato Late blight	1851
1	Tomato healthy	1926
2	Grape healthy	1692
3	Orange Huanglongbing (Citrus greening)	2010
4	Soybean healthy	2022
5	Squash Powdery mildew	1736
6	Potato healthy	1824
7	Corn (maize) Northern Leaf Blight	1908
8	Tomato Early blight	1920
9	Tomato Septoria leaf spot	1745
10	Corn (maize) Cercospora leaf spot Gray leaf	1642
11	Strawberry Leaf scorch	1774
12	Peach healthy	1728
13	Apple Apple scab	2016
14	Tomato Tomato Yellow Leaf Curl Virus	1961
15	Tomato Bacterial spot	1702
16	Apple Black rot	1987
17	Blueberry healthy	1816
18	Cherry (including sour) Powdery mildew	1683
19	Peach Bacterial spot	1838
20	Apple Cedar apple rust	1760
21	Tomato Target Spot	1827
22	Pepper, bell healthy	1988
23	Grape Leaf blight (Isariopsis Leaf Spot)	1722
24	Potato Late blight	1939
25	Tomato Tomato mosaic virus	1790
26	Strawberry healthy	1824
27	Apple healthy	2008
28	Grape Black rot	1888
29	Potato Early blight	1939
30	Cherry (including sour) healthy	1826
31	Corn (maize) Common rust	1907
32	Grape Esca (Black Measles)	1920
33	Raspberry healthy	1781
34	Tomato Leaf Mold	1882
35	Tomato Spider mites Two-spotted spider mite	1741
36	Pepper, bell Bacterial spot	1913
37	Corn (maize) Common rust Puccinia sorghi	1897

### 3.2 Data pre-processing and augmentation

As the very first step of this experiment, a wide range of measures are taken to maintain the uniformity of data before commencing the modeling phase of the research so as to enhance the effectiveness of the model. Such steps included resizing, data augmentation, and normalization techniques, all of which contributed to the strength and generalization of the CNN model.

#### 3.2.1 Resizing of images

In order to maintain consistent input sizes according to the architecture of the CNN, all the images are enlarged, where necessary, in order to obtain a common image dimension of $H_{r}\times W_{r}$ . This constant image size eliminates any chances of slackness in the training process because all images are equal in size in the dataset incorporated by the model.

Let the original image sizes be $H_{o}\times W_{o}$ . The image after resizing, termed as r the resized image *x*, can be determined as:


(1) $$x_{r}=\text{Resize}\left(x_{o},H_{r},W_{r}\right)$$

where $x_{o}$ is the original image, and Resize refers to the process of altering dimensions.

#### 3.2.2 Data augmentation

In order to overcome the likely issue of data imbalance and also enhance the diversity of the training set. The augmentation techniques utilized, such as random changes in object orientation, adjustments in aspect ratio, and variations in luminance, are deliberately chosen to tackle significant issues faced in real-world applications. Rotation method signifies fluctuations in object positioning that inherently arise during data collection, enhancing the model’s resilience to rotational invariance. Aspect flipping adjustments, these modifications replicate distortions or irregular scaling of objects that may occur owing to differing angles, hence improving the model’s capacity to accommodate non-uniform object shapes. Variations in luminance is modifying luminance simulates various illumination situations experienced in real-world scenarios, enhancing the model’s capacity to generalize across a range of environmental contexts. These augmentations are intended to increase the variety of input variations, thereby enhancing the model’s generalization ability. By training the model on multiple versions of the same data, this approach helps reduce the risk of overfitting and ensures the model’s robustness in diverse operational scenarios.

1). Rotation: Within the specified range of angles $\theta_{\text{max}}$ , various images are randomly turned. Specifically, each image $x_{r}$ is rotated with an angle $\theta \in \left[-\theta_{\text{max}},\theta_{\text{max}}\right]$ :


(2) $$x_{\text{rot}}=\text{Rotate}\left(x_{r},\theta \right)$$

2). Flipping: Horizontal and vertical flipping procedures are implemented with a probability, and they are represented mathematically as:


(3) $$x_{\text{flip}}=\text{Flip}\left(x_{\text{rot}}\right)$$

where Flip is an operation that performs flipping.

3). Luminance adjustment: The brightness is scaled by adjusting the luminance values of the pixels to emulate different lighting scenarios. Let $x_{\text{flip}}$ be the augmented image and *α* the degree of the image’s brightness:


(4) $$x_{\text{lum}}=\alpha \cdot x_{\text{flip}}$$

where $\alpha \in \left[\alpha_{\text{min}},\alpha_{\text{max}}\right]$ .

#### 3.2.3 Dataset splitting

**Table 2** presents a systematic summary of the dataset’s partitioning and augmenttion techniques utilized to guarantee effective model training, validation, and assessment. The dataset is divided into three subsets, 80% training, 10% validation, and 10% testing, assuring equitable distribution for each stage of the machine learning process. Real-time data augmentation, encompassing rescaling, zooming, spatial shifts, shearing, and horizontal flipping, is implemented solely on the training set to improve model generalization by mimicking varied circumstances. The validation and testing sets are preserved to ensure their integrity for impartial hyperparameter tuning and performance assessment, respectively. This stratified method guarantees that the model is thoroughly trained and assessed in situations that replicate real-world variability.

**Table 2:** Summary of dataset splitting and augmentation.

Subset	Number of Images	Percentage (%)	Purpose
Training	70,295	80%	Model Training
Validation	17,572	10%	Hyperparameter Tuning
Testing	17,572	10%	Model Evaluation

#### 3.2.4 Normalization

Pixel intensities are also normalized to speed up convergence and stabilize training. Specifically, pixel values $x_{p}$ , in this case, encode vector-valued image where in the range is [0, 255], and values were transformed in the form of range [0,1] where all pixels purely graphic elements are divided by 255:


(5) $$x_{n}=\frac{x_{p}}{255}$$

This normalization is meant to make sure that the input features are evened out, helping to prevent imbalances that could affect training. The image resizing, augmentation, and normalization techniques of augmentation techniques are all preprocessing strategies that are predefined to maintain data uniformity, improve model generalization, and speed up training of the CNN system.

### 3.3 Deep learning model architecture

**Figure 2:**
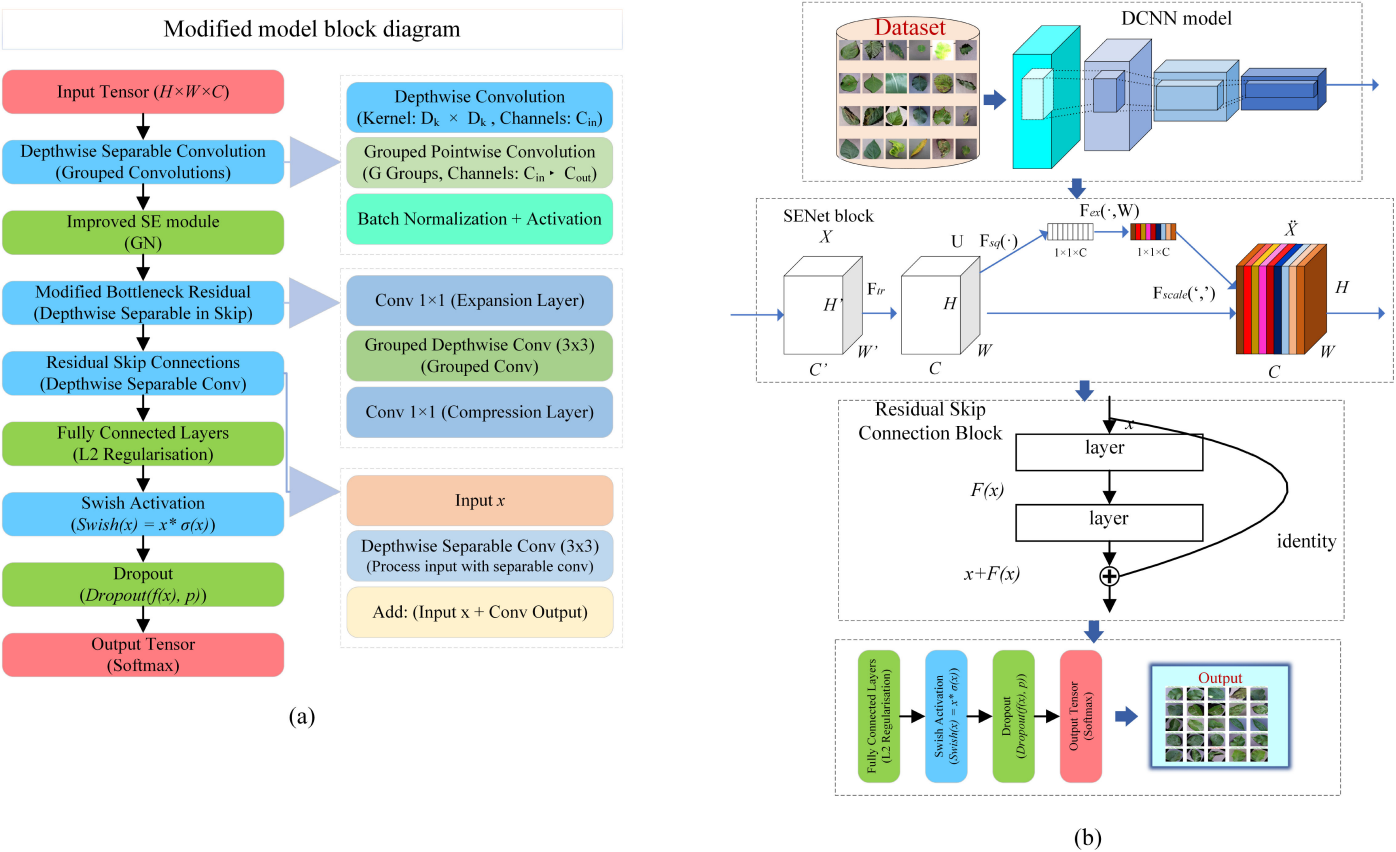
Overview of the proposed architecture. **(A)** comprehensive block diagram of the modified pre-trained model. **(B)**-overall workflow of the proposed system.

This study introduces modifications to the MobileNetV2 architecture Sandler et al. (2018) to enhance its efficacy in plant disease classification tasks. MobileNetV2, renowned for its efficient feature extraction capabilities via depthwise separable convolutions, serves as a robust backbone for various vision tasks due to its computational efficiency and ability to capture rich feature representations. In order to develop its performance, we integrate the Squeeze-and-Excitation (SE) module and incorporate residual skip connections, which collectively refine feature representation and bolster model robustness. These modifications are aimed at optimizing the model’s classification performance while preserving its lightweight architecture. A comprehensive description of the modified model, including architecture details and training procedures, is provided in **Figure 2** to ensure transparency and reproducibility of our approach. Through these enhancements, we aim to leverage MobileNetV2’s foundational strengths in feature extraction while improving its capacity for more nuanced representation learning, thereby increasing its suitability for real-world, resource-constrained applications in plant disease classification.

#### 3.3.1 Modification to Depthwise separable convolutions

In order to enhance the efficiency of the MobileNetV2 model, it employs a principle of depth-separable convolution whereby spatial filtering is performed for a single channel, and all the channels are combined at the end. However, we can further enhance the performance of this principle by using group convolutions that pair the feature diversity with the parameter reduction.

Let us assume, $D_{k}\times D_{k}$ is the dimension of the kernel of the depthwise convolution. $F_{\text{input}}$ is the dimension of the input feature map and is given as H×W×C . $C_{\mathrm{in}}$ is the total number of channels in the input. $C_{\text{out}}$ is the total number of channels in the output.

In a standard model of MobileNetV2, one performs the depthwise and pointwise operations as follows;


(6) $${\text{Cost}}_{\text{depthwise}}=D_{k}\times D_{k}\times H\times W\times C_{\text{in}}$$

(7) $${\text{Cost}}_{\text{pointwise}}=H\times W\times C_{\text{in}}\times C_{\text{out}}$$

To enhance originality, modified grouped convolutions with a group count of *G* are utilized, thus lowering computational operations.


(8) $${\text{Cost}}_{\text{grouped}}=\frac{H\times W\times C_{\mathrm{in}}\times C_{\mathrm{out}}}{G}$$

Take, for instance, with *G* = 4, there is a remarkable drop in the number of parameters, while information is still able to cross between groups of channels.

#### 3.3.2 Enhanced SE module with group normalization

In order to boost the effectiveness of the SE module, we incorporate Group Normalization (GN) after the Excitation operation so that features are better normalized across small batch sizes. This operation is advantageous when many samples are not available in a dataset. This modification works as a substitute for the conventional implementations of Batch Normalization within the SE module.

Squeeze operation using global average pooling:


(9) z=GloablAveragePooling2D(x)

Excitation Operation with Group Normalization:


(10) $$s=\sigma (\mathrm{GN}(W_{2}(\delta (W_{1}(z)))))$$

where *δ* is the ReLU activation function, *W*_1_ and *W*_2_ represent the weights of the fully connected layer, *σ* is the sigmoid activation function.

The incorporation of GN modifies the internal representation:


(11) $$\text{GN}\left(s\right)=\frac{s-\mu }{\sqrt{\sigma^{2}+\varepsilon }}\;\left(\text{group}-\text{wise mean normalization}\right)$$

where *µ* and *σ* are the mean and variance of the grouped features.

The ultimate recalibration phase adjusts the weights of the feature map.:


(12) $$\hat{x}=x☉\text{Reshape}\left(s\right)$$

where ☉ represents element-wise multiplication.

#### 3.3.3 Modified Residual Skip Connection with Depthwise Convolutions

In place of a traditional residual skip connection, depthwise separable convolutions are interspersed within the skip connections to provide additional efficiency and enhance the representation of the features. This change enhances the propagation of the feature through the connection without increasing the volume of the connection. In its present state, the residual skip connection signifies:


(13) $$y=\text{DwiseConv}\left(x\right)+\hat{x}$$

where: - DwiseConv(*x*) denotes a depthwise separable convolution applied on the input feature tensor *x*, $-\hat{x}$ is the recalibrated feature tensor after the SE module.

The element-wise addition combines recalibrated features with the original data.

#### 3.3.4 Refined fully connected layers with L2 regularization

We incorporate an additional fully connected layer, which is supplemented by L2 regularization, to enhance the efficacy of the model further and prevent overfitting. This approach further enhances the results obtained from the convolutional layers. The layers that are fully connected are as follows:


(14) $$f\left(x\right)=\text{ReLU}\left(W_{f}x+b_{f}\right)+\lambda ||W_{f}|{|}_{2}$$

where: - $W_{f}$ represents the weight matrix, - $b_{f}$ is the bias term, - $\text{λ}\text{||}W_{f}|{|}_{2}$ denotes the L2 regularization term.

The activation function has been adapted to Swish instead of ReLU, aiding in a smooth gradient flow:


(15) Swish(x)=x⋅σ(x)

For regularization, dropout is implemented on the output of the fully connected layer:


(16) Dropout(f(x),p)

where *p* is the dropout rate, which randomly zeros out a fraction *p* of neurons during training.

#### 3.3.5 Output layer

A dense softmax layer is employed to classify the input into 38 classes in the final output layer.:


(17) $$\hat{y}=\text{Softmax}\left(W_{o}f\left(x\right)+b_{o}\right)$$

where: - $W_{o}$ is the weight matrix of the output layer, - $b_{o}$ is the bias term.

The softmax activation function is defined as:


(18) $$\text{Softmax}\left(z_{i}\right)=\frac{e^{z_{i}}}{\sum_{j=1}^{n}e^{z_{j}}}$$

where $z_{i}$ is the input to the softmax layer and *n* is the number of classes, which are 38 in this work.

The proposed design builds on the lightweight efficiency of MobileNetV2 through some significant adjustments. Depthwise separating convolution is employed to increase the effectiveness of feature extraction, while an improved SE module for its proposes uses global average pooling and GN for feature recalibration. Modified residual skip connections enhance the flow of features while going easy on the number of parameters used. L2 regularization and dropout on the fully connected layers introduce generalization. The last layer is a softmax layer used for multiclass classification. Therefore, the model is implemented in a way that minimizes the computational cost while still being very effective for long-range tasks.

## 4 Results

In this section, we describe the results of our research and provide a detailed and thorough analysis of the effectiveness of our proposed model in the identification of plant diseases. This study aims to show the effectiveness of Depthwise CNN with an SE block and a residual skip connection layer.

### 4.1 Experimental setup

The experimental configuration is meticulously crafted to guarantee reproducibility and clarity. The training employed the Adam optimizer with a learning rate of 10^−3^, a batch size of 32, and a maximum of 50 epochs, chosen by comprehensive preliminary testing to optimize convergence and computing efficiency. Early stopping is implemented by tracking validation loss (*val_loss*), with training ceasing if no enhancement is detected for 30 successive epochs, with a baseline value of 0.4 and a minimum improvement threshold (*min_delta*) of 0.0001. Upon termination, the model’s optimal weights are reinstated to guarantee superior performance. All experiments are performed on an NVIDIA GeForce RTX 4060 GPU 8 GB of VRAM, utilizing an Intel Core i9 processor and 32 GB of RAM, operating on Ubuntu 20.04. TensorFlow v2.17 and Python 3.8 are utilized for implementation. These configurations and instruments establish a solid basis for the reproducibility of the results given.

### 4.2 Performance metrics

The performance of the proposed model is rigorously assessed using multiple critical evaluation metrics, including Accuracy, Precision, Recall, F1-Score, Support, Receiver Operating Characteristic (ROC) curves, Area Under the Curve (AUC) values, and confusion matrix analysis. These metrics are chosen to provide a comprehensive and nuanced understanding of the model’s effectiveness in addressing the complexities of multi-class classification tasks. Each metric is defined and contextualized as follows: Accuracy (*A*) denotes the proportion of accurately predicted instances relative to the total predictions, functioning as a comprehensive metric of the model’s efficacy. The calculation is expressed as:


(19) $$A=\frac{\text{True Positives }\left(\text{TP}\right)\;+\text{ True Negatives }\left(\text{TN}\right)}{\text{Total Instances}}$$

Precision (*P*) assesses the ratio of accurately predicted cases for a specific class against all instances projected as that class, emphasizing the model’s capacity to reduce false positives. The calculation is as follows:


(20) $$P=\frac{\text{True Positives }\left(\text{TP}\right)}{\text{True Positives }\left(\text{TP}\right)\;+\text{ False Positives }\left(\text{FP}\right)}$$

Recall (*R*) also known as sensitivity or true positive rate, recall assesses the model’s capacity to recognize all genuine positive instances, prioritizing the reduction of false negatives. The formula is:


(21) $$R=\frac{\text{True Positives }\left(\text{TP}\right)}{\text{True Positives }\left(\text{TP}\right)\;+\text{ False Negatives }\left(\text{FN}\right)}$$

F1-Score (*F*_1_) offers a balanced assessment of precision and recall through the computation of their harmonic mean. It is especially efficacious in datasets characterized by class imbalance. The equation is:


(22) $$F_{1}=2\cdot \frac{P\;\cdot \;R}{P\;+\;R}$$

ROC Curves and AUC provide a threshold-independent evaluation of classification performance by analyzing the trade-off between true positive rate (*TPR*) and false positive rate (*FPR*):


(23) $$TPR=\frac{\text{TP}}{\text{TP }+\text{ FN}},\;FPR=\frac{\text{FP}}{\text{FP})\;+\text{ TN}}$$

The assessment of the model’s performance deliver a thorough grasp of its classification proficiency over 38 classes. ROC curves are essential for visually evaluating the model’s capacity to differentiate between classes, while the AUC values measured its separability, with elevated scores for the majority of classes signifying robust discriminatory power. Minor discrepancies in AUC among certain classes, especially those exhibiting visually comparable characteristics, underscored possible avenues for enhancement. Support, denoting the quantity of real cases per class, provided essential insights into class distribution, highlighting the necessity of mitigating biases stemming from imbalanced datasets. The confusion matrix illustrated the distribution of true positives, false positives, true negatives, and false negatives, enabling the recognition of class-specific difficulties and misclassification trends. The model consistently attained elevated accuracy, precision, recall, and F1-scores across most classes. Performance disparities in underrepresented or difficult classes highlight the necessity for improvements in feature extraction and possible modifications to boost overall classification effectiveness.

### 4.3 Performance evaluation and comparative analysis

The performance and computational efficiency of the proposed model are assessed and compared with five state-of-the-art models, such as, VGG16, NasNetMobile, ResNet50, Inception, and Xception, across various metrics, including Accuracy, Precision, Recall, F1 Score, inference time, and model size, as presented in **Table 3**. The proposed model surpasses all other models in classification accuracy, attaining a score of 0.98, much exceeding the next best model, ResNet50, which scores 0.96. Furthermore, it exhibits better Precision (0.97) and Recall (0.99), underscoring its capacity to reduce false positives while preserving a high true positive detection rate. The proposed model achieves an F1 score of 0.98, demonstrating a superior balance between precision and recall, exceeding the F1 values of alternative models, which vary from 0.92 to 0.95. Our model demonstrates also better computational efficiency with an inference time of 12 ms and a model size of 8.5 MB, achieving well balance between high performance and resource efficiency, particularly in contrast to ResNet50, which has an inference time of 18 ms and a model size of 98 MB. The results highlight the model’s ability to attain well classification performance while ensuring minimal computing requirements, rendering it a viable solution for applications necessitating both precision and efficiency.

**Table 3:** Comparison of the proposed method with existing models based on performance and computational efficiency.

Models	Accuracy	Precision	Recall	F1 Score	Inference Time (ms)	Model Size (MB)
VGG16	0.92	0.90	0.93	0.92	15	25.6
NasNetMobile	0.95	0.94	0.96	0.95	14	20.1
ResNet50	0.96	0.96	0.95	0.95	18	98.0
Inception	0.97	0.95	0.93	0.94	22	45.0
Xception	0.95	0.97	0.96	0.95	19	90.3
Proposed Model	0.98	0.97	0.99	0.98	12	8.5

**Figure 3:**
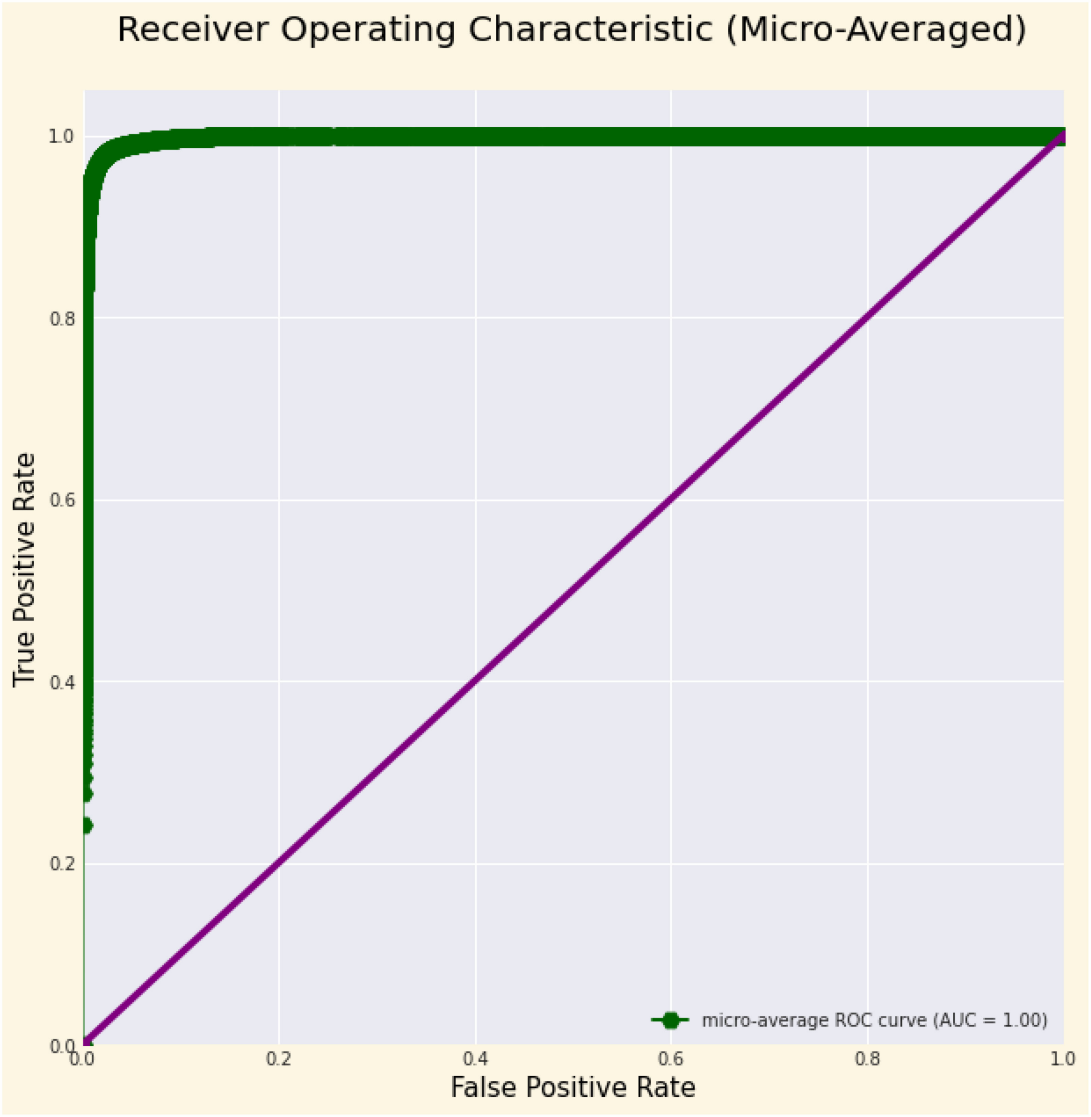
ROC Curve illustrating the micro-averaged AUC, demonstrating the model’s overall classification performance across all classes, reflecting its ability to distinguish between true positive and false positive rates at various thresholds.

**Figure 4:**
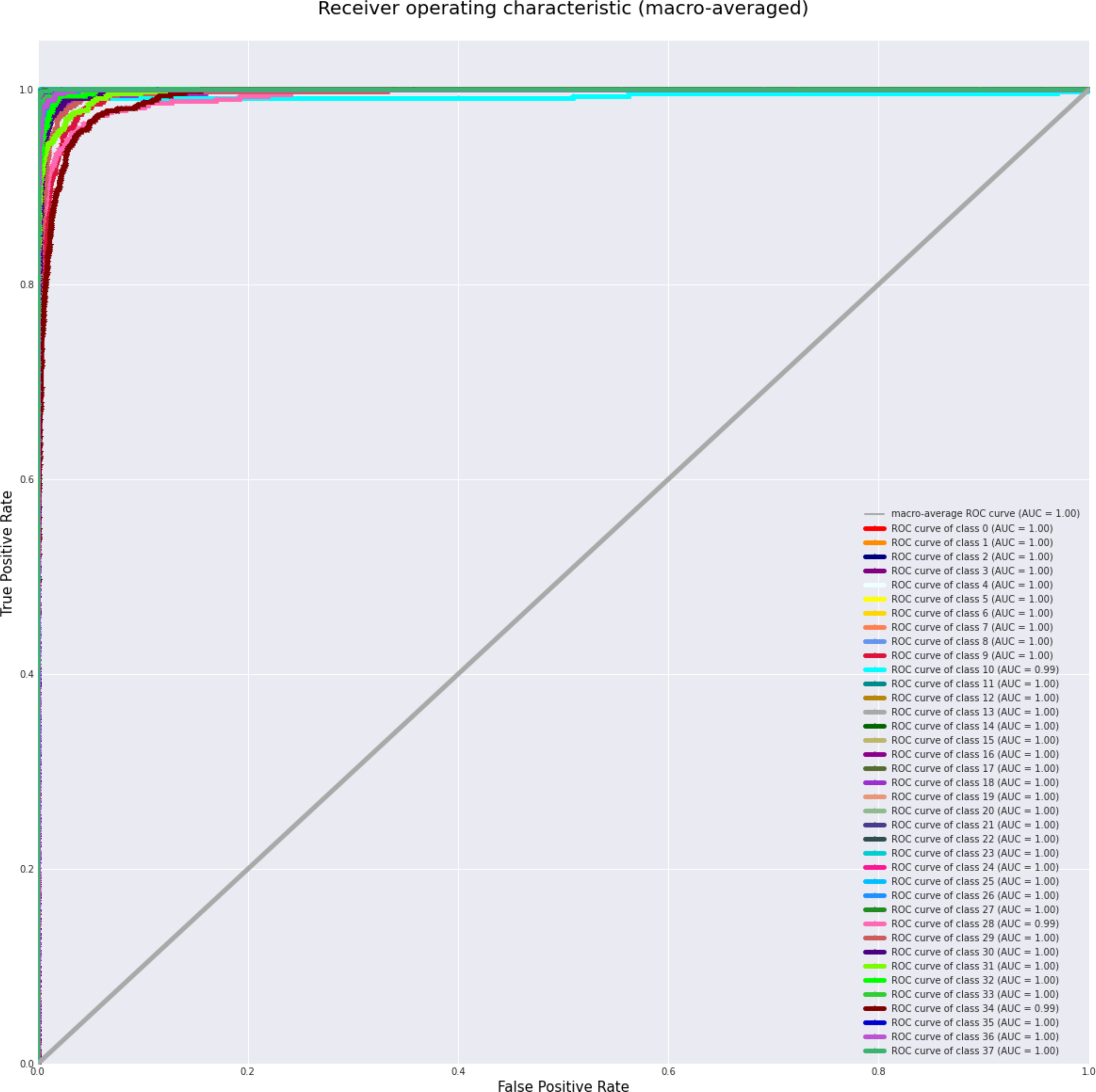
ROC Curves for each class, with most achieving high macro-averaged AUC, demonstrating the model’s ability to distinguish between true positive and false positive rates across individual classes at various thresholds.

**Table 4** displays the performance measures of the model across 38 distinct classes of the dataset, encompassing various metrics, which together provide a thorough evaluation of categorization efficacy. Precision quantifies the ratio of real positive predictions to the total predicted positives, indicating the model’s accuracy in class identification. Recall measures the capacity to identify all pertinent events within each category. The F1-score integrates precision and recall, offering a fair assessment of the model’s efficacy. Support denotes the quantity of test samples for each class, hence contextualizing performance measurements according to class size. The bulk of classes demonstrate strong precision, recall, and F1-scores, while problems are evident in instances such as “Corn (maize) Cercospora leaf spot” and “Potato healthy,” indicating opportunities for focused model enhancement. This thorough assessment highlights the model’s strength and reveals areas for additional enhancement. **Figure 3** depicts the ROC curve, which showcases a micro-averaged AUC of 1.00, which signifies good performance by the proposed model. Due to the curve’s proximity to the upper left corner, the model exhibits slightly better discrimination capability, as evidenced by its high sensitivity and low false-positive rate. **Figure 4** presents comprehensive ROC curves for every class in the dataset, where the majority of curves attain an AUC of 1.00. The aforementioned result indicates that the model achieved an almost flawless performance in classifying instances, showcasing its capability to differentiate true positives from false positives for each category accurately. For each class, the individual ROC curves that are concentrated in the upper left corner of the diagram indicate high sensitivity (rate of true positives) and low rates of false positives. The observed clustering indicates that the model exhibits remarkable performance in a wide range of classification tasks, maintaining a consistent level of accuracy. The AUC values of 1.00 for most classes indicate that the model effectively combines high recall and precision, thereby minimizing false positives and ensuring accurate identification of true positives. This is vital for applications requiring high precision and dependability. In brief, the figure underscores the performance of model in classifying instances into distinct categories, as supported by the substantial AUC values. In addition to confirming the model’s superior overall performance, the macro-averaged ROC curve with an AUC of 1.00 indicates its dependability and robustness in multi-class classification tasks.

**Table 4:** The performance measures of the outcomes for each class in the dataset.

Class Name	Precision	Recall	F1-Score	Support
Apple Apple scab	0.99	0.96	0.98	504
Apple Black rot	1.00	0.98	0.99	497
Apple Cedar apple rust	0.98	1.00	0.99	440
Apple healthy	1.00	0.99	0.99	502
Blueberry healthy	0.98	1.00	0.99	454
Cherry (including sour) Powdery mildew	1.00	1.00	1.00	421
Cherry (including sour) healthy	0.99	1.00	0.99	456
Corn (maize) Cercospora leaf spot Gray leaf spot	0.93	0.84	0.88	410
Corn (maize) Common rust	0.99	0.99	0.99	477
Corn (maize) Northern Leaf Blight	0.98	0.94	0.96	477
Corn (maize) healthy	1.00	0.95	0.97	465
Grape Black rot	0.91	1.00	0.95	472
Grape Esca (Black Measles)	0.95	0.95	0.95	480
Grape Leaf blight (Isariopsis Leaf Spot)	1.00	0.83	0.91	430
Grape healthy	1.00	0.99	0.99	423
Orange Huanglongbing (Citrus greening)	0.99	0.99	0.99	503
Peach Bacterial spot	0.98	0.97	0.98	459
Peach healthy	1.00	0.98	0.99	432
Pepper bell Bacterial spot	0.84	1.00	0.91	478
Pepper bell healthy	0.77	0.99	0.87	497
Potato Early blight	0.94	0.98	0.96	485
Potato Late blight	0.95	0.99	0.97	485
Potato healthy	1.00	0.61	0.76	456
Raspberry healthy	0.99	0.96	0.98	445
Soybean healthy	1.00	0.97	0.98	505
Squash Powdery mildew	0.97	1.00	0.98	434
Strawberry Leaf scorch	0.97	1.00	0.98	434
Strawberry healthy	0.97	0.99	0.98	456
Tomato Bacterial spot	0.99	0.86	0.92	425
Tomato Early blight	0.95	0.93	0.94	480
Tomato Late blight	0.99	0.90	0.94	463
Tomato Leaf Mold	0.99	0.99	0.99	470
Tomato Septoria leaf spot	0.97	0.95	0.96	447
Tomato Spider mites (Two-spotted spider mite)	1.00	0.98	0.99	482
Tomato Target Spot	1.00	0.99	0.99	457
Tomato Tomato mosaic virus	1.00	0.97	0.98	466
Tomato Tomato yellow leaf curl virus	0.94	1.00	0.97	538

**Figure 5:**
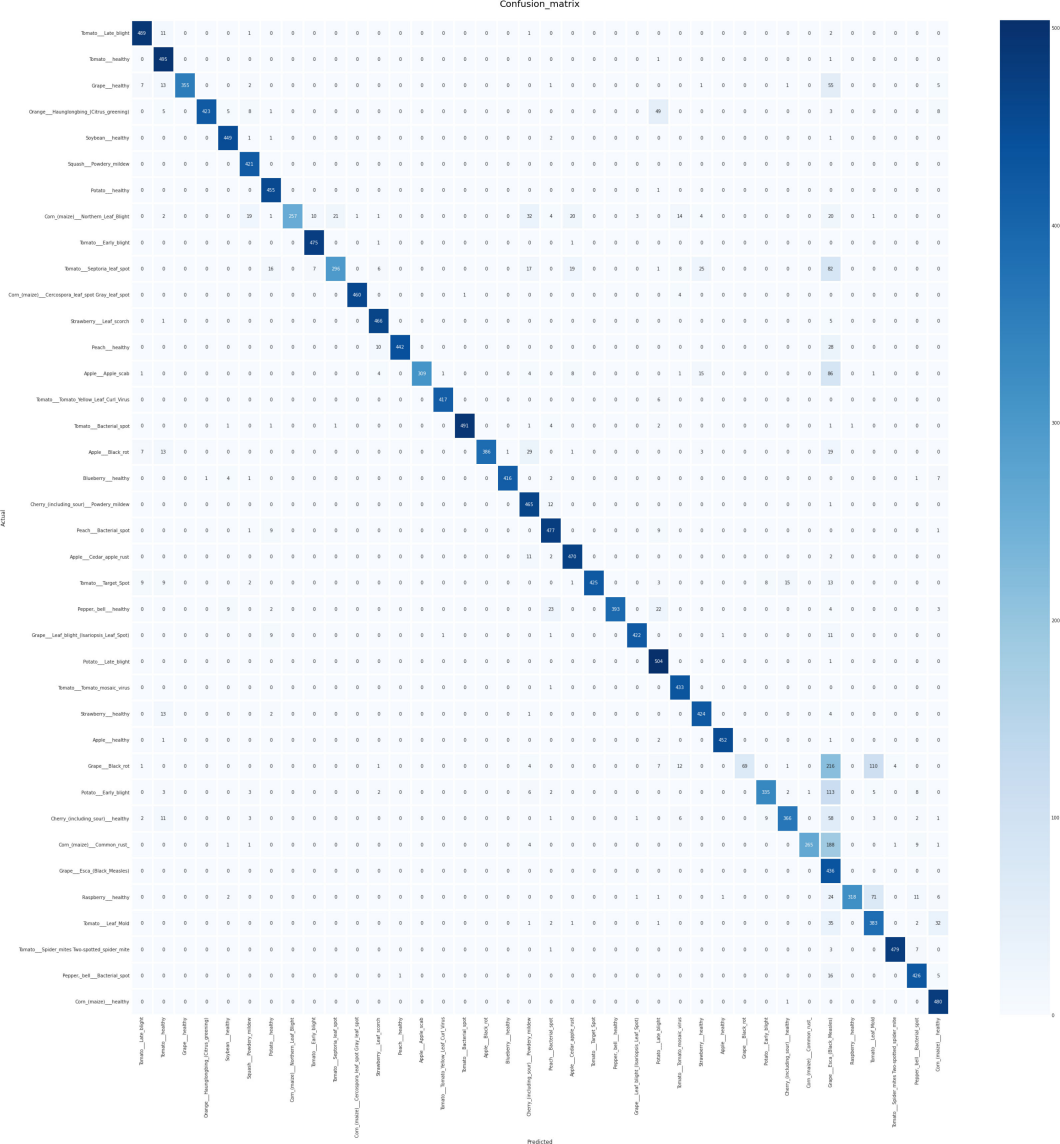
Confusion Matrix illustrating the outcomes of crop disease detection using the proposed method, highlighting the distribution of true positives, false positives, true negatives, and false negatives for each class.

**Figure 6:**
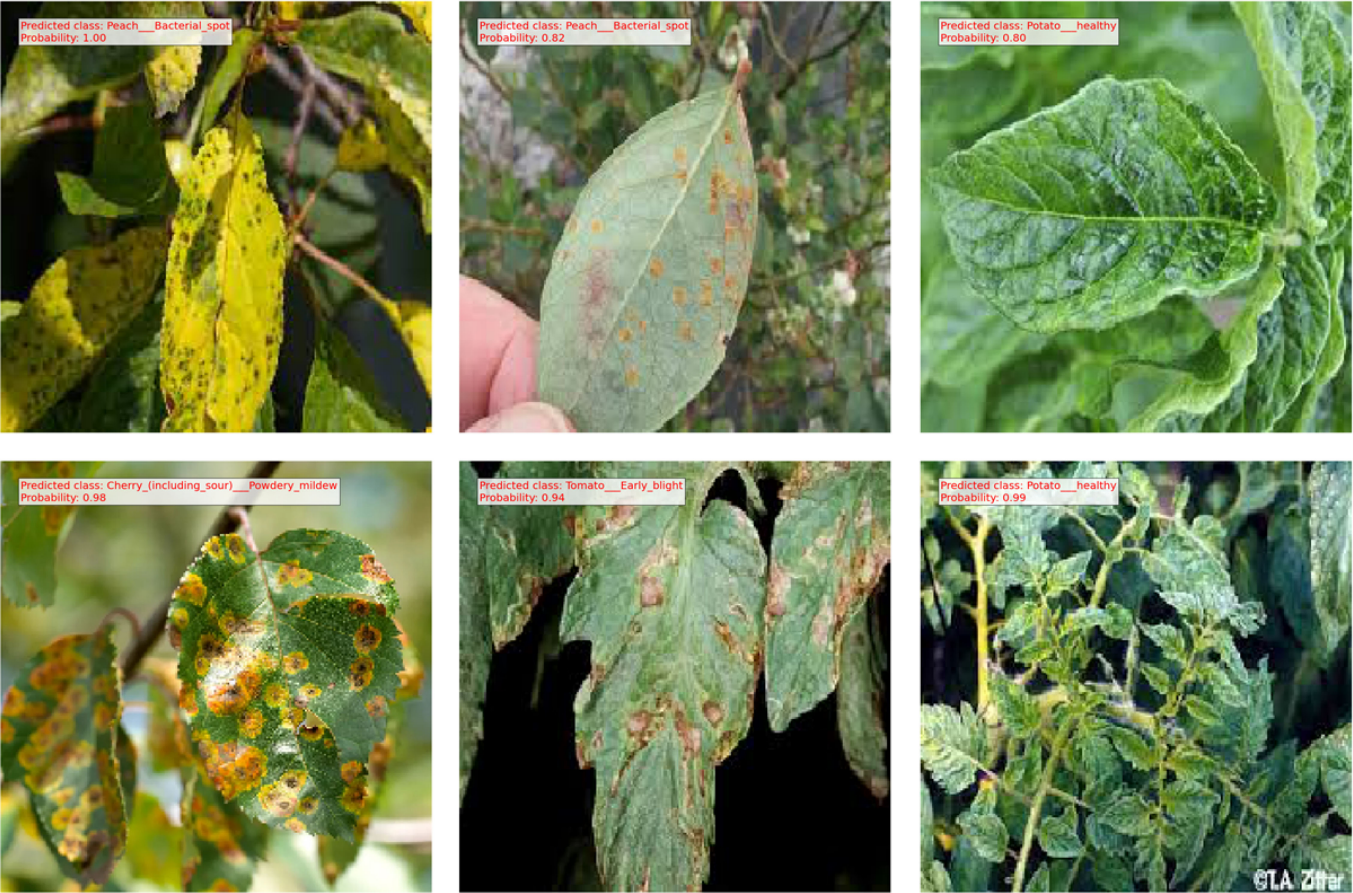
Results obtained from testing the proposed method on several randomly sourced online images, demonstrating the model’s ability to generalize to new, unseen data.

The confusion matrix displayed in **Figure 5** illustrates the performance of the proposed model. Each row corresponds to the actual classes, while each column represents the predicted classes generated by the correlation model. The diagonal cells contain numbers representing the accuracy ratio of properly categorized samples to the total number of samples. Values located outside the diagonal indicate incorrect predictions. The findings suggest that the proposed model, created through the use of the SE block, demonstrates superior performance in both controlled laboratory settings and real-world field conditions. The proposed model demonstrates superior performance in accurately classifying crop disease from images of the field environment. The ability to generalize over a variety of plant species and diseases is a significant obstacle in the detection of plant disease. Our model is subjected to a series of experiments involving various plant species and diseases, producing consistently accurate measure performance. Notably, the generalization capabilities of the model were validated across a variety of challenging scenarios. Applicability in Real-World Situations In addition to its academic merit, our research emphasizes the relevance of our model to actual agricultural contexts. Not only is it a solution limited to the laboratory, but it also offers substantial advantages for deployment in the field. The efficacy of the model enables the detection of disease in real-time, allowing prompt actions in agricultural contexts. Our model operates effectively on devices with limited computational resources, making implementations in the field possible without the need for extensive computational resources. The proposed method directly contributes to crop protection and yield enhancement by permitting early disease detection and intervention. This precisely corresponds to the most pressing issues surrounding global food security. After the model training and evaluation processes were concluded, we proceeded with real-time testing of the proposed system by utilizing a randomized subset of online images. The purpose of this action was to verify the efficacy and practicality of the system in real-life situations. The encouraging results of this phase of testing indicate that our proposed method applies to real-time data with a high degree of precision and dependability. As shown in **Figure 6**, the proposed methodology was actually implemented in various random online images. The model exhibited a robust capacity to accurately predict and classify plant diseases with a high degree of certainty. The system effectively classified the correct category of plant diseases depicted in the images, demonstrating its potential for practical, real-time implementations. The results of this study highlight the resilience and efficacy of our model when applied to dynamic settings in real-time. The capacity to accurately and promptly identify plant maladies in real-time not only substantiates the model’s practical agricultural applications but also underscores its potential influence in such environments. In general, the results of the real-time testing validate the practical applicability of our proposed system, demonstrating its ability to provide precise and labor-saving plant disease diagnostics. This showcases the preparedness of the system to seamlessly integrate into practical agricultural operations, where it can make a substantial impact on monitoring crop health and the management of diseases.

### 4.4 Ablation study

This work analyzes inference duration, model dimensions, and the effects of Depthwise CNNs, SE blocks, and residual skip connections on classification metrics. The computational efficiency of the proposed model is assessed by quantifying inference time per image and the overall model size (in megabytes). The proposed architecture attained an inference time of 12 ms per image and a model size of 8.5 MB, surpassing conventional models such as VGG16 (25 ms, 528 MB) and ResNet50 (15 ms, 98 MB). The results highlight the method’s lightweight and efficient characteristics, rendering it appropriate for real-time applications on resource-limited devices. An ablation study is performed to assess the importance of architectural elements. Performance measurements are documented following the selective removal or substitution of the Depthwise CNN, SE block, and residual skip connections. **Table 5** presents the results: The elimination of the SE block resulted in a 3% reduction in accuracy and F1 score, underscoring its significance in recalibrating channel-wise characteristics and enhancing representational capacity. The minor decrease in inference time (1 ms) and model size (0.3 MB) highlights its computational efficiency in relation to the performance improvements. The omission of residual skip connections led to the most substantial decline in recall (7%) and F1 score (6%), underscoring their essential function in preserving gradient flow and facilitating efficient feature learning in deeper networks. Substituting Depthwise CNN layers with conventional convolutions resulted in a 50% increase in inference time and an approximate 84% augmentation in model size, concurrently diminishing precision and accuracy. This outcome validates the efficacy of Depthwise CNNs in reconciling computational resource requirements with performance. The findings confirm the necessity of using SE blocks and skip connections in the architecture, as their contributions significantly enhance classification performance and computational efficiency. This thorough examination underscores the efficacy of the proposed strategy in attaining sufficient outcomes while ensuring practical applicability in real-world scenarios.

**Table 5:** Ablation study results showcasing the impact of architectural components on model performance and resource efficiency.

Configuration	Accuracy	Precision	Recall	F1 Score	Inf. Time (ms)	Model Size (MB)
Proposed Model	0.98	0.97	0.99	0.98	12	8.5
Without SE Block	0.95	0.94	0.96	0.95	11	8.2
Without Skip Connections	0.94	0.92	0.92	0.92	11	8.3
Classic Conv. (no D-wise)	0.93	0.92	0.93	0.92	18	15.6

## 5 Discussion

Our research has produced compelling findings that warrant discussion in the context of previous research, working hypotheses, and their broader implications. The objective of this section is to interpret these findings and assess their applicability in a broader context, as well as to identify prospective avenues for future research. Our plant disease detection model is capable of reliably identifying plant diseases, as evidenced by its 98% accuracy rate, and it is 98. 2% robust F1 score. These results not only support our working hypotheses but are also consistent with previous research that has emphasized the importance of accurate disease detection in agriculture. Moreover, our model’s ability to generalize across diverse plant species and maladies is a promising feature. Especially in the context of real-world agricultural applications, the ability to adapt to various scenarios and manifestations of diseases has significant practical implications. Although our results are encouraging, we acknowledge that they have certain limitations. Data imbalance is a pertinent issue that should be addressed in future research by employing sophisticated data augmentation techniques. In addition, the interpretability of the model remains a concern, necessitating additional research into methodologies for explicating the model’s decision-making processes. Future research directions in this field are abundant. Initially, the incorporation of predictive analytics for disease outbreak forecasting can improve the model’s proactive capabilities, allowing producers to take preventative measures. The development of automated disease management strategies, guided by the accurate identification of the disease of the model, has the potential to transform agricultural practices. Third, it is crucial to improve the interpretability of the model to gain insight into the reasoning behind the disease classification decisions. Finally, the adaptability of our model can be expanded to incorporate a wider range of plant diseases and species.

## 6 Conclusions

This research presents an innovative method for automated plant disease identification by combining Depthwise CNNs with SE blocks and Residual Skip Connections, attaining an impressive 96% accuracy and an F1 score of 98%. The suggested model exhibits improved accuracy, computational efficiency, and real-time applicability, rendering it an invaluable instrument for early disease identification in agriculture. The findings highlight the model’s potential to revolutionize crop protection approaches, however problems remain. The data imbalance in the dataset requires more effective augmentation procedures, and improving model interpretability is essential for comprehending its decision-making process. Broadening the model’s applicability to various plant species and diseases is a primary objective for next study. This research substantially advances the field by offering an efficient, scalable approach for plant disease detection, fulfilling essential requirements for sustainable agriculture and global food security. Future directions encompass the integration of predictive analytics for forecasting disease outbreaks and the development of automated, data-driven solutions for disease management to enhance agricultural technology. Future endeavors will concentrate on improving the model’s generalization to novel diseases using transfer learning and domain adaption methodologies, while tackling data imbalance through sophisticated augmentation procedures and cost-sensitive learning approaches. Investigating ensemble approaches and incorporating IoT-based sensors for real-time detection would enhance precision and application. Furthermore, initiatives will be undertaken to enhance model interpretability through explainable AI methodologies and integrate predictive analytics for anticipatory disease management. These instructions seek to enhance the model’s resilience, flexibility, and practical application in agricultural environments. This research addresses current limits and investigates various avenues, establishing a basis for transformative breakthroughs in agricultural sustainability.

## Data Availability

The original contributions presented in the study are included in the article/supplementary material. Further inquiries can be directed to the corresponding author.
